# Supplementary material for: Influence of Caffeic and Caftaric Acid, Fructose, and Storage Temperature on Furan Derivatives in Base Wine
Source: Molecules. 2022 Nov 15;27(22):7891. doi: 10.3390/molecules27227891 (PMC9692380; doi:10.3390/molecules27227891)
Supplement: Supplementary file 1 [file molecules-27-07891-s001.zip › molecules-2020399-supplementary.pdf]

## Supplementary Materials

# Influence of caffeic and caftaric acid, fructose, and storage temperature on furan derivatives in base wine.

Jacob Medeiros [1], Shufen Xu [2], Gary J. Pickering [1,2,3,4] and Belinda S. Kemp [1, 2*]

Table S1. Concentrations of hydroxycinnamic acids and hydroxycinnamates reported in sparkling wines aged for 1–2 years post-disgorgement at cellar temperature.

| **Concentration Ranges in Sparkling Wine (mg/L)** | | | | | |
|---|---|---|---|---|---|
| | Blanc de Blanc | | Blanc de Noir | | |
| | MPX[1] | Chardonnay | Trepat | Monastrell | Pinot Noir |
| ***Hydroxycinnamic acids*** | | | | | |
| ***Trans*-caffeic acid** | 0.48[a]–1.90[c] | 1.3[b]–1.6[b] | 0.93[d] | 0.20[d] | 1.71[b]–2.0[b] |
| ***Trans-p*-coumaric acid** | 0.24[a]–1.50[c] | 0.34[b]–0.62[b] | 0.33[d] | 0.13[d] | 0.16[b]–0.54[b] |
| ***Trans*-ferulic acid** | 0.60[c] | 0.07[b]–0.40[b] | 0.35[d] | 0.16[d] | 0.36[b]–0.41[b] |
| ***Hydroxycinnamates*** | | | | | |
| ***Trans*-caftaric** | 3.82[a]–20.25[d] | 29[b]–31[b] | 2.17[d] | 2.57[d] | 11[b]–29[b] |
| ***Trans*-coutaric** | 1.01[a]–5.40[c] | 0.36[b]–31[b] | 0.32[d] | 0.59[d] | 0.11[b]–0.46[b] |
| ***Trans*-fertaric** | 0.68[d]–1.22[a] | 0.70[b]–0.90[b] | 0.10[d] | 0.17[d] | 0.54[b]–0.70[b] |

[1] MPX = Macabeu, Xarel-lo, Parellada varietal blend. [a] [1], [b] [2], [c] [3], [d] [4]

S.1. Wine sample preparation

Samples were prepared using five separate stock solutions, using the same base wine for all treatments and weighing each compound before their additions to the base wine (Denver Instrument Model P-214, Bohemia, New York, USA). Solution 1 included untreated base wine, and Solution 2 (12 g/L fructose) was prepared by mixing 12 g of pure fructose (D-fructose (≥ 99%) in 1000 mL of base wine. Solution 3 (10 mg/L caffeic acid) was made by adding 5 mg of pure caffeic acid (CAS 331-39-5, ≥ 98%) directly to 500 mL of base wine, and Solution 4 (10 mg/L caftaric acid) was made in the same fashion by adding 5 mg of caftaric acid (CAS 67879-58-7, ≥ 97%) to 500 mL of base

wine. Finally, Solution 5 (10 mg/L caffeic acid and 10 mg/L caftaric acid) was prepared by adding 5 mg of pure caffeic acid and 5 mg of pure caftaric acid to 500 mL of base wine. Treatments were made in 50 mL (Narrow Mouth HDPE) plastic screw-top bottles (VWR® International, Radnor, Pennsylvania, USA). This was achieved by combining the appropriate solutions to yield the level of addition required as follows: F + CAFT treatment was created by combination of 25 mL of Solution 2 with 25 mL of Solution 4, yielding a mixture containing 6 g/L added fructose and 5 mg/L added caftaric acid. The space above the liquid was sparged with argon gas before being closed with the cap to prevent oxidation. Bottles were stored at 30 °C undisturbed in an insulated, temperature-controlled room set to 30 °C for 90 days. Those stored at 15 °C were placed in the wine cellar at the Cool Climate Oenology and Viticulture Institute (CCOVI) (maintained at 15 °C) for 90 days.

S.2. Total Hydroxycinnamic Acid (HCA) Estimation and Degree of Browning

Across temperature conditions, variable trends in total HCA estimation were observed, with significant differences in HCA estimated content between treatments and storage temperature conditions ($K$ (DF 15) = 61.7, $p < 0.0001$). Samples stored at 30 °C increased in total HCA content over the 90 day period, but those stored at 15 °C either did not change or decreased (Figure S1a & 2b). Following the storage period, CAFE treatment samples retained a greater total HCA estimation compared to the CAFT treatments ($\Delta$ 0.140 A.U., $p < 0.0001$) (Figure S1a & b).

Brown pigment estimation was also determined to be significantly different based on treatment and storage condition ($K$ (DF 15) = 45.2, $p < 0.0001$). Following the 90 days of

storage, a significant degree of brown pigment accumulation was observed across all 30 °C treatments compared to the 15 °C samples ($p < 0.05$). No differences were determined based on treatment effect (Figure S2b).

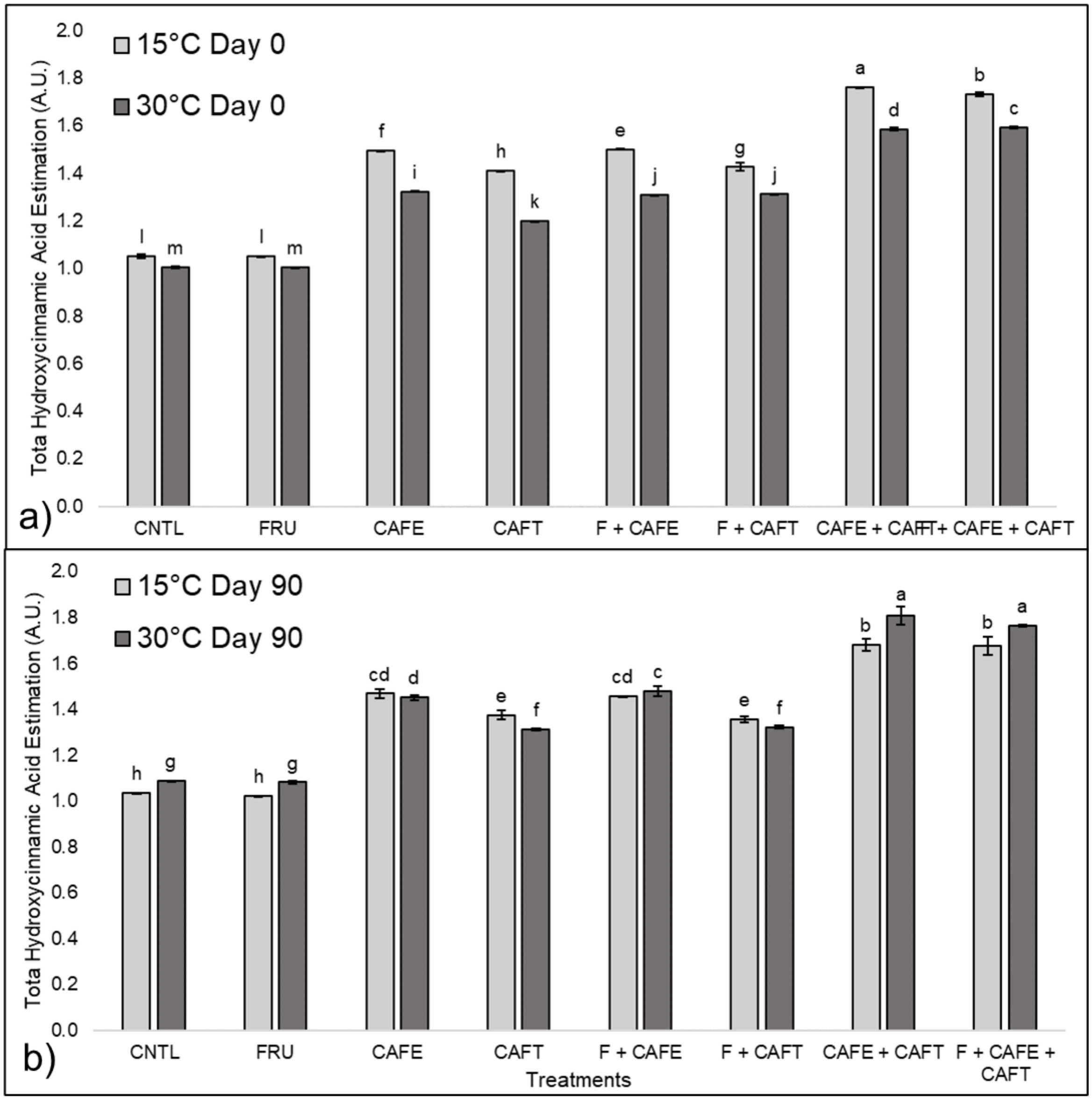


Figure S1a & b. Total hydroxycinnamic acid (HCA) estimation (A.U) in base wine samples at Day 0 (a) and Day 90 (b) stored at 15 and 30 °C. Treatment codes: CNTL (no addition), FRU (6 g/L fructose), CAFE (5 mg/L caffeic acid), CAFT (5 mg/L caftaric acid), F + CAFE (6 g/L fructose + 5 mg/L caffeic acid), F + CAFT (6 g/L fructose + 5 mg/L caftaric acid), CAFE + CAFT (5 mg/L caffeic acid + 5 mg/L caftaric acid), and F + CAFE + CAFT (6 g/L fructose + 5 mg/L caffeic acid + 5 mg/L caftaric acid). Error bars represent the standard deviation of sample means ($n$ = 4). Multiple comparison of treatment means was carried out via the Kruskal-Wallis test, using Treatment and Storage Temperature as independent categorical variables, followed by the Conover-Iman procedure. Different letters represent the difference between means as determined by the pairwise comparisons of sample means at $p < 0.05$.

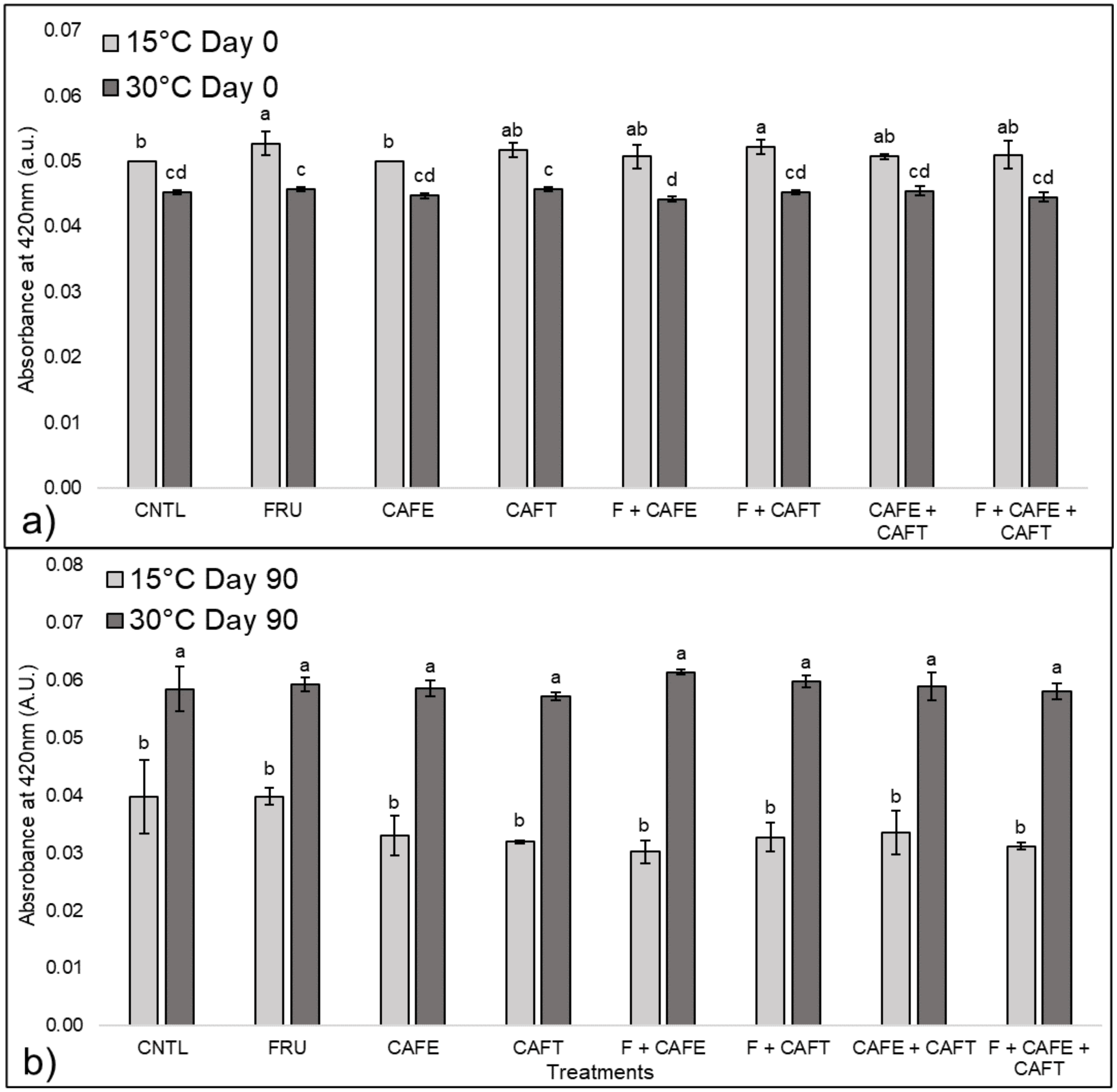


Figure S2a & b. Degree of browning (A.U) in base wine samples at Day 0 (a) and Day 90 (b) stored at 15 and 30 °C. Treatment codes: CNTL (no addition), FRU (6 g/L fructose), CAFE (5 mg/L caffeic acid), CAFT (5 mg/L caftaric acid), F + CAFE (6 g/L fructose + 5 mg/L caffeic acid), F + CAFT (6 g/L fructose + 5 mg/L caftaric acid), CAFE + CAFT (5 mg/L caffeic acid + 5 mg/L caftaric acid), and F + CAFE + CAFT (6 g/L fructose + 5 mg/L caffeic acid + 5 mg/L caftaric acid). Error bars represent the standard deviation of sample means ($n$ = 4). Multiple comparison of treatment means was carried out via the Kruskal-Wallis test, using Treatment and Storage Temperature as independent categorical variables, followed by the Conover-Iman procedure. Different letters represent the difference between means as determined by the pairwise comparisons of sample means at $p < 0.05$.

## References


1. Serra-Cayuela, A.; Jourdes, M.; Riu-Aumatell, M.; Buxaderas, S.; Teissedre, P.L.; López-Tamames, E. Kinetics of Browning, Phenolics, and 5-Hydroxymethylfurfural in Commercial Sparkling Wines. *J. Agric. Food Chem.* **2014**, *62*, 1159–1166, doi:10.1021/jf403281y.

2. Chamkha, M.; Cathala, B.; Ronique Cheynier, V.Ä.; Douillard, R. Phenolic Composition of Champagnes from Chardonnay and Pinot Noir Vintages. *J. Agric. Food Chem.* **2003**, *51*, 3179–3184, doi:10.1021/jf021105j.

3. Bosch-Fusté, J.; Sartini, E.; Flores-Rubio, C.; Caixach, J.; López-Tamames, E.; Buxaderas, S. Viability of Total Phenol Index Value as Quality Marker of Sparkling Wines, "cavas". *Food Chem.* **2009**, *114*, 782–790, doi:10.1016/j.foodchem.2008.10.013.

4. Pozo-Bayo, M.A.; Hernandez, M.T.; Martin-Alvarez, P.J.; Polo, M.C. Study of Low Molecular Weight Phenolic Compounds during the Aging of Sparkling Wines Manufactured with Red and White Grape Varieties. *J. Agric. Food Chem.* **2003**, *51*, 2089–2095, doi:10.1021/jf021017z.
